# Lung Inflammation in STING-Associated Vasculopathy with Onset in Infancy (SAVI)

**DOI:** 10.3390/cells11030318

**Published:** 2022-01-18

**Authors:** Clémence David, Marie-Louise Frémond

**Affiliations:** 1 Université de Paris, Imagine Institute, Laboratory of Neurogenetics and Neuroinflammation, 24 Boulevard du Montparnasse, 75015 Paris, France; 2 Paediatric Immunology-Hematology and Rheumatology Department, Hôpital Necker-Enfants Malades, APHP.Centre-Université de Paris, 24 Boulevard du Montparnasse, 75015 Paris, France

**Keywords:** interferons, nucleic acid sensing, STING-associated vasculopathy with onset in infancy

## Abstract

STING-associated vasculopathy with onset in infancy (SAVI) is a type I interferonopathy caused by gain-of-function mutations in *STING1* encoding stimulator of interferon genes (STING) protein. SAVI is characterized by severe inflammatory lung disease, a feature not observed in previously described type I interferonopathies i.e., Mendelian autoinflammatory disorders defined by constitutive activation of the type I interferon (IFN) pathway. Molecular defects in nucleic acid metabolism or sensing are central to the pathophysiology of these diseases, with such defects occurring at any step of the tightly regulated pathway of type I IFN production and signaling (e.g., exonuclease loss of function, RNA-DNA hybrid accumulation, constitutive activation of adaptor proteins such as STING). Among over 30 genotypes, SAVI and COPA syndrome, whose pathophysiology was recently linked to a constitutive activation of STING signaling, are the only type I interferonopathies presenting with predominant lung involvement. Lung disease is the leading cause of morbidity and mortality in these two disorders which do not respond to conventional immunosuppressive therapies and only partially to JAK1/2 inhibitors. In human silicosis, STING-dependent sensing of self-DNA following cell death triggered by silica exposure has been found to drive lung inflammation in mice and human models. These recent findings support a key role for STING and nucleic acid sensing in the homeostasis of intrinsic pulmonary inflammation. However, mechanisms by which monogenic defects in the STING pathway lead to pulmonary damages are not yet fully elucidated, and an improved understanding of such mechanisms is fundamental to improved future patient management. Here, we review the recent insights into the pathophysiology of SAVI and outline our current understanding of self-nucleic acid-mediated lung inflammation in humans.

## 1. Introduction

Type I interferonopathies are a sub-group of Mendelian autoinflammatory diseases characterized by an overactivation of the type I interferon (IFN) pathway [1]. Under normal circumstances, IFNs are potent antiviral cytokines produced by cells when they sense foreign nucleic acids [2]. In type I interferonopathies, monogenic defects in the recognition process of self versus non-self nucleic acids are the cornerstone of the pathogenesis [3,4,5]. The concept of type I interferonopathy was defined by Yanick J. Crow in 2011 to encompass this group of inborn errors of immunity [6], and implied that blocking the type I IFN pathway would alleviate the patient symptoms. A few years later, the observation of an improvement in disease status of patients with type I interferonopathies treated by blockers of the type I IFN pathway (i.e., JAK inhibitors) [7] supported the hypothesis of a direct role of type I IFNs in the pathogenesis of these diseases. Along with the increasing number of genotypes described—from 7 to almost 40 in 10 years—the clinical phenotype extended from mainly neurologic and cutaneous manifestations [8] to articular, renal, pulmonary, and other organ involvement. In 2014, the molecular study of unrelated children affected with cutaneous vasculopathy and interstitial lung disease (ILD) defined a new Mendelian disease: STING-associated vasculopathy with onset in infancy (SAVI) [9,10]. SAVI is caused by gain-of-function mutations in the *STING1* (previously named *TMEM173*) gene encoding STING (stimulator of interferon genes). *STING1* gain-of-function mutations enhanced type I IFN pathway activation and treatment of lymphocytes from SAVI patients with JAK1/2 inhibitors reduced the constitutive phosphorylation of STAT1 [9], thereby defining SAVI as a novel type I interferonopathy. The description of an inflammatory lung disease caused by monogenic mutations in *STING1* opened the field of nucleic acid sensing and STING homeostasis in lung pathology. We propose here a review of what is known about pulmonary involvement in SAVI, what remains to be understood, and how improvement of our knowledge about STING gain-of-function pathogenesis in lung disease can be relevant for other human pathologies.

## 2. STING Signaling

### 2.1. STING and Type I Interferons

**Figure 1:**
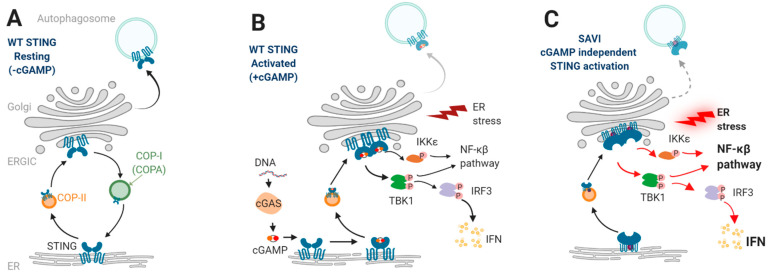
STING Signaling. (**A**,**B**) Upon DNA sensing by cGAS, cGAMP binds to wild-type (WT) STING. STING trafficks then to the Golgi and undergoes post-translational modifications. STING activation in the Golgi leads to type I interferon (IFN) production, NF-κβ pathway activation, and cell death induction through endoplasmic reticulum (ER) stress. STING trafficking is normally controlled through retro-transport to the ER via COPA and by trafficking to the autophagosome, where it is degraded. (**C**) When affected by a gain-of-function mutation, STING is constitutively activated in the Golgi in a cGAMP-independent manner and enhances constitutive type I IFN production through IRF3 phosphorylation and inflammatory cytokine production by activating the NF-κβ pathway. STING constitutive activation in the Golgi also induces ER stress, which can further lead to cell death. Finally, reduction of STING clearance by autophagy is described in the context of STING gain-of-function. Adapted from Frémond et al., J Clin Immunol, 2021. Created with biorender.com, accessed on 26 November 2021, last accessed on 13 January 2022.

Detection of intracellular foreign nucleic acids is a major pathway of innate immunity in most organisms. Recognition of cytoplasmic double-stranded DNA (dsDNA) relies on the cyclic GMP-AMP synthase (cGAS)–STING pathway (Figure 1A,B). Regardless of its origin, dsDNA binds to and activates cGAS, which produces endogenous cyclic dinucleotide (CDN) 2′3′ cyclic GMP–AMP (cGAMP) [11]. cGAMP is then detected by the endoplasmic reticulum (ER)-resident transmembrane protein STING. Although cGAMP is the only known mammalian CDN, bacteria release similar CDNs that can also bind directly to STING and trigger its activation [12]. Of note, intercellular transfer of cGAMP to bystander cells can amplify inflammatory responses by activating STING in these cells, independently of nucleic acid sensing [13]. Upon binding to cGAMP, STING undergoes conformational changes that lead to oligomerization of STING dimers [14]. This allows the release of STING dimers from their anchoring protein and their subsequent translocation from the ER through the ER–Golgi intermediate compartment (ERGIC) to the Golgi. STING trafficking from the ER to the Golgi depends on its incorporation into the coatomer protein complex (COP)-II [15] and is regulated by different proteins, such as iRhom2 and YIPF5, that, respectively, facilitates trafficking [16] and supports STING sorting into the COP-II vesicle [17]. In the Golgi, STING undergoes several post-translational modifications, including palmitoylation [18,19]. By promoting its dimerization-mediated autophosphorylation [19], STING then activates TANK-binding kinase 1 (TBK1), which, in turn, phosphorylates STING to form STING–TBK1 complex and recruit IFN regulatory factor 3 (IRF3) [20]. TBK1 then phosphorylates IRF3, enabling its nuclear translocation to promote type I IFN expression [21]. After activation in the Golgi, STING supposedly translocates to the lysosome, where it is degraded [22]. STING homeostasis also relies on its retro-transport from the Golgi to the ER through COP-I [23]. The crucial role of COP-I in terminating STING activation has been recently discovered by the elucidation of a monogenic auto-inflammatory disease caused by heterozygous dominant negative mutations in *COPA*, encoding the subunit α of COP-I [24,25]. Indeed, mutant COPA induced accumulation of STING to the Golgi and overaction of the type I IFN pathway. These recent findings highlight the role of COPA in preventing chronic immune activation through STING [7,26,27].

### 2.2. STING Enhances NF-κβ Pathway

The incremental discoveries on STING biology have highlighted that STING function is not solely limited to type I IFN pathway [28]. This is illustrated by the fact that STING protein is expressed in species that do not possess IFN system [29,30]. In HEK293T cells, expression of cGAS–STING also increased *NF-κB* promoter activity [26,27], and in mouse embryonic fibroblast cells (MEF), STING activation triggered NF-κβ-mediated cytokine response (e.g., tumor necrosis factor [TNF] and IL-6), mainly through TBK1 [31]. However, TBK1 is not mandatory for NF-kβ-mediated cytokine production [32]. In fact, it has been recently evidenced that TBK1 acts redundantly with Ikβ kinase ε (IKKε) to drive NF-kβ signaling upon STING activation [32]. In addition, NF-kβ phosphorylation was significantly higher under TBK1/IKKε inhibition than IRF3 phosphorylation. Considering the role of the NF-kβ pathway in promoting auto-immunity and auto-inflammation [33], these findings suggest that cGAS–STING-mediated NF-kβ inflammatory pathway also represents a valuable therapeutic target to effectively ameliorate the inflammatory STING-mediated disorders.

### 2.3. STING and Autophagy

In recent years, interactions between the autophagy machinery and the cGAS–STING pathway have been extensively studied. Autophagy is a catabolic process, whereby cytoplasmic components (damaged or long-lived organelles and/or proteins) are enveloped in double membrane vesicles (autophagosomes) that subsequently fuse with lysosomes for degradation and/or recycling. In contrast, selective autophagy permits cell survival by targeting specific proteins and organelles for removal, such as damaged mitochondria (mitophagy), to maintain quality and product energy [34]. The first demonstration of STING involvement in autophagy has been evidenced in the context of mycobacterium tuberculosis infection in mice [35]. Indeed, extracellular mycobacterial DNA was sensed by cGAS–STING pathway to activate type I IFN response and also autophagy [35]. To be delivered to autophagosomes, bacteria need to be ubiquitinated, a process in which STING activation seems to play a key role [36]. Although not fully understood, STING-mediated autophagy appears to be type I IFN- and TBK1-independent [37]. It may instead rely on trafficking of STING vesicles through the ERGIC, where autophagosomes are formed by the ERGIC membrane and target cytosolic DNA for degradation by the lysosome. It is suggested that STING then traffics to lysosomes, where it is degraded [38]. Since a role for autophagy has been described in lung pathology [39], understanding how STING gain-of-function can perturbate autophagy biology in lungs would be relevant for the comprehension of pulmonary disease in SAVI.

### 2.4. STING and Cell Death

Cell death is a senescence-independent process that is important to prevent oncogenic transformation. There are several interactions between cGAS–STING pathway and cellular death processes [40]. Emerging evidence suggests that ER stress secondary to STING activation can induce cell death. Indeed, STING exit from the ER triggers calcium efflux, thereby inducing ER stress and the unfolded protein response (UPR), which can subsequently lead to cell death in T cells [41]. Moreover, upon STING activation, phosphorylated IRF3 can translocate to mitochondria, where it causes Bax-mediated cell death [21,42]. Finally, STING trafficking to the lysosome can also result in lysosome membrane permeabilization and, subsequently, lysosome-mediated cell death in human myeloid cells [43]. Of note, an intrinsic and IFN-independent antiproliferative action of activated STING has been described in T cells and is reminiscent of the T cell proliferation defect seen in SAVI [44] and the profound lymphopenia observed in certain mouse models discussed below [45].

## 3. Sting-Associated Vasculopathy with Onset in Infancy (SAVI)

Of importance, SAVI, a life-threatening condition caused by gain-of-function mutations in *STING1*, has highlighted the need for controlling STING signaling.

### 3.1. Historical Description

In 2014, Liu et al. reported six unrelated children who presented with systemic inflammation, ILD, and cutaneous vasculopathy [9]. They identified three heterozygous missense mutations (V155M, N154S, and V147L) transmitted de novo or as a somatic mosaicism in one case. They observed a strong transcriptional IFN-stimulated gene (ISG) signature in the peripheral whole blood of four explored patients, thereby suggesting a gain-of-function mechanism. This was confirmed using an in vitro model of HEK293T cells overexpressing non-mutant and mutant STING constructs, where *IFNB1* reporter activity was elevated in cells transfected with any of the three mutant constructs. STING is present in most cells (proteinatlas.org). Of importance, the authors observed that STING was expressed in endothelial cells from cutaneous biopsy samples of patients and in type II alveolar epithelial cells, bronchial epithelium, and alveolar macrophages from lung tissue sections [9].

The same year, Jeremiah et al. reported a three-generation family with four affected members harboring the V155M mutation [10] responsible for lung fibrosis, recurrent fevers, and autoimmunity in the proband. This report highlighted a possible intrafamilial clinical variability in SAVI since adult-onset symptoms and milder disease were observed in the grandfather of the proband. Functional data confirmed constitutive activation of type I IFN pathway in patients’ cells and in cellular models.

Since 2014, more than 70 SAVI cases have been reported in the literature. The phenotype has expanded, including arthritis, thyroiditis [46], glomerulonephritis [47], or cerebrovascular involvement [48] and revealed the possibility of an autosomal recessive inheritance [49].

The description of the novel type I interferonopathy SAVI [1,2,50] thus indicated that *STING1* gain-of-function mutations led to a severe Mendelian inflammatory ILD. These findings suggest a potential role for STING homeostasis in ILD.

### 3.2. SAVI Pathogenesis

**Figure 2:**
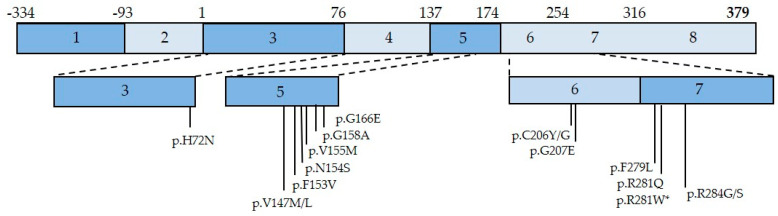
Schematic Representation of STING Protein Encoded by *STING1* Gene and Localization of Disease-Causing Mutations. * indicates homozygous mutations.

The two most frequent *STING1* mutations i.e., V155M and N154S, located in the same mutation cluster (including other mutants, such as G166E, V147M or H72N [51]), are assumed to induce STING constitutive activation by promoting the 180° rotation of the ligand-binding domain, thus resulting in STING oligomerization, independently of any interaction with its ligand cGAMP [52]. Other mutations in the second disease-causing mutation cluster (C206, R281, or R284) are thought to suppress the auto-inhibition of STING oligomerization [14]. All the mutations reported so far in the literature are presented in Figure 2. Interestingly, there is no evident phenotype–genotype correlation in SAVI [52].

As presented above, STING regulates several pathways, which may all be perturbed by a monogenic dysfunction of the protein (Figure 1C). Gain-of-function heterozygous mutations in *STING1* were shown to lead to constitutive activation of STING. Indeed, in SAVI, STING is abnormally localized to the Golgi, where it induces IFN signaling pathway [53]. Transcriptomic analysis of the whole blood of three SAVI patients compared with healthy controls showed differential expression of 119 genes, including mainly ISGs, but also genes involved in other innate and adaptative immune processes [54]. Beyond type I IFNs, the potential pathogenic effect of type III IFNs (IFN-λ1-4), which are mostly expressed in epithelium, is also suspected in SAVI. Indeed, they have been shown to be detrimental to respiratory epithelium after viral infection in the case of chronic secretion [55]. Of note, STING has been described as driving IFN- λ1 production in human cells after detection of viral DNA [50].

In addition to IFN production, *STING1* gain-of-function mutations are also thought to trigger NF-κβ pathway. SAVI patients displayed high C-reactive protein levels [1,2,9] and upregulated NF-κβ-related protein (e.g., IL6) expression in peripheral blood mononuclear cells (PBMCs) or whole blood [1,9,56]. In addition, *STING1* gain-of-function mutations markedly enhanced NF-κβ activation in luciferase reporter systems [57]. However, the relative role of NF-κβ activation in disease pathogenesis requires further studies.

As spontaneous cell death has been observed in T cells, monocytes, and endothelial cells from SAVI patients, a link between *STING1* gain-of-function mutations and cell death has been suggested to participate in the pathogenesis of SAVI [44]. At least, this could explain the T cell deficiency observed in patients, with low counts of memory CD8+ T cells and impaired T cell proliferation in response to antigens. To our knowledge, autophagy has never been evaluated in SAVI patients’ cells, but this pathway may also contribute to disease pathogenesis.

Although our understanding of cellular and molecular consequences of *STING1* gain-of-function mutations is improving, mechanisms underlying the life-threatening lung disease in SAVI are not yet fully deciphered.

### 3.3. Lung Involvement in SAVI Patients

**Figure 3:**
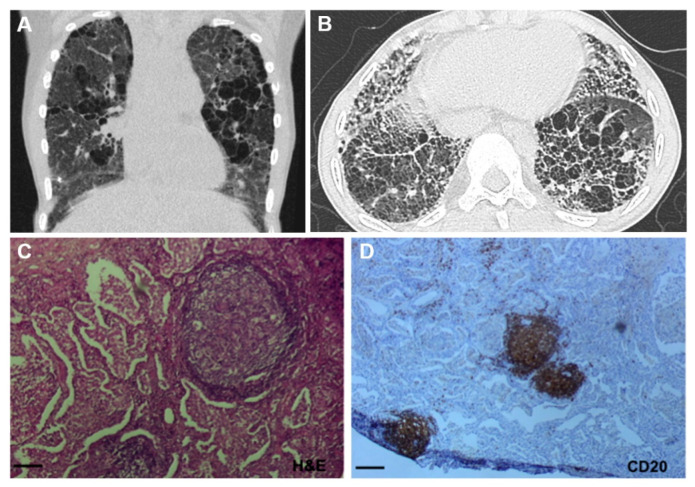
Representative Lung Imaging and Pathology in SAVI. (**A**,**B**) Coronal and axial image of a chest computed tomography (CT) scan from a SAVI patient showing interstitial lung disease with ground glass opacities, cysts, and septal wall thickening. (**C**,**D**) Lung tissue section biopsy from a SAVI patient showing lymphoid infiltrate consisting mainly of CD20+ cells. Original magnification: ×40 (**C**; scale bars, 100 µm), ×10 (**D**; scale bars, 400 µm) Adapted from Jeremiah et al., J Clin Invest, 2014 and Frémond et al., J Allergy Clin Immunol Pract, 2021.

Lung involvement leads to high morbidity and mortality in SAVI [47]. More than 75% of patients described so far presented with ILD [52]. Alveolar hemorrhage has been reported in few patients [48]. Half of the patients harboring ILD presented with radiologic or histologic evidence of lung fibrosis [52], even at a very young age [58]. Respiratory symptoms in SAVI can be insidious and are not specific (chronic cough, exertional dyspnea, and hemoptysis) (Table 1). Nail clubbing has been described in some patients. Pulmonary function tests showed mainly a restrictive syndrome with diffusion impairment, sometimes associated with hyperinflation, but also obstruction or a mixed lung function impairment [58]. High-resolution chest computed tomography (CT) can evidence different patterns suggestive of ILD: ground-glass opacities, septal thickening, or reticulation cysts. Features of radiological fibrosis include honeycombing, traction bronchiectasis, and lung volume reduction (Figure 3). Chest CT can also show signs evocative of alveolar hemorrhage, such as ground glass opacities and/or focal alveolar condensation. Finally, intrathoracic lymphadenopathies can be found in SAVI patients [52]. Of interest, radiological lesions of ILD are more frequently asymmetrical than in other ILDs related to connective tissue diseases [58]. Bronchoalveolar lavage (BAL) fluid frequently revealed an increased number of cells (termed as alveolitis) with a non-specific pattern (either lymphocytic, neutrophilic, or mixed) [48]. In some cases, BAL was normal. Lung biopsy histopathological analysis mainly showed interstitial fibrosis and/or fibrosis without particular specificity. Lymphoid follicles with germinal center organization and the presence of CD20+ B cells and T-cell infiltration have also been observed (Figure 3) [52]. Some cases displayed pulmonary vasculitis [48] or desquamative interstitial pneumonia without further precision [59].

### 3.4. SAVI Lung Disease Pathogenesis

STING is broadly expressed, including in lung cells (proteinatlas.org, last accessed on 13 January 2022). The relative contribution of intrinsic lung cells and cells derived from the hematopoietic system to the pulmonary inflammation and fibrosis seen in SAVI is unknown.

To elucidate SAVI pathogenesis, three mouse models of the two most common SAVI-associated mutations have been developed. The first model of a N153S knock-in mouse, recapitulating the human N154S mutations, was published by Warner et al. in 2017 [56]. The N153S mice were generated using CRISPR/Cas9 on C57BL/6N mice. The generated mice globally recapitulated the observed human phenotype as they showed lung inflammation, T cell cytopenia, and skin ulceration. Histopathological evaluation of the mice lungs revealed chronic perivascular inflammation with heterogeneous immune cell infiltration and thrombosis in the lung blood vessels. However, no evidence of lung fibrosis was initially found. To be noted, this mouse model showed pleural effusion [56]. When limiting the use of antibiotics in the next generation of these mice, features of lung fibrosis were found in their biopsies [60]. Lung disease in the N153S mice appeared to be T-cell-mediated, since *Rag1^−/−^* STING N153S mice, which lack T cells and mature B cells, exhibited no histological evidence of lung disease and *Tcrβ^−/−^* STING N153S mice developed only very mild lung disease [60,61]. A disruption in the development of second lymphoid organs and, consequently, a reduction of innate lymphoid cells in mice were also demonstrated [62]. These results suggest a central role of hematopoietic-derived cells in the lung disease of N153S mice. One major difference from human SAVI was the absent or mild expression of ISG in N153S mice, pointing to IFN-independent pathogenesis.

Bouis et al. developed a heterozygous V154M mouse model, recapitulating the human V155M mutation, using CRISPR/Cas9 technology on C57BL/6N mice. This model mainly had immunological features with severe combined immunodeficiency (SCID) phenotype, together with lung and kidney inflammation [63]. Of note, 45% of the mice presented with alveolar or perivascular inflammation in the lung, but no fibrosis was noted. Due to the severity of immunodeficiency, absence of skin disease, lung fibrosis, or arthritis, this mouse model also differed from the human phenotype. Moreover, the SCID phenotype was (almost completely) IFN-independent.

In contrast to the systems described above, a third mouse model, involving the engineering of human STING1 with the gain-of-function N154S mutation in hematopoietic murine cells, demonstrated a phenotype bearing greater similarity to the human disease state in terms of IFN signaling status and skin disease [64]. The human *STING-N154S* mutant cDNA was inserted downstream from the murine Vav1 to obtain hematopoietic-cell-specific expression. Specifically, these mice displayed significant increased IFN-alpha levels in serum and a paw vasculopathy that was rescued when crossing onto an Ifnar1 null background. However, no lung disease or inflammation was observed. The relevance of these mouse models to the human phenotype remains uncertain, although they highlight the possibility of non-IFN-driven pathogenesis in SAVI.

Since members within the same family can present with variable clinical manifestations, ranging from early pulmonary fibrosis to mild ILD developing in adulthood [10], the question of additional factors driving lung disease is of major interest. The explanation for this observation remains unknown, but might be determined by environmental triggers (e.g., infection and vaccination), additive or protective genetic factors, or epigenetic modifications [65].

### 3.5. Futures Perspectives to Decipher SAVI Lung Disease Pathogenesis

Several factors may explain why mouse models do not appear to fully recapitulate human lung disease nor be the appropriate model to study SAVI pathogenesis. Firstly, differences exist between human and mice lungs. In addition to an obvious difference in terms of organism size, lung development, function properties related to surfactant homeostasis, and lung vasculature differ between mice and humans [66,67,68]. As a representative example, the bronchial circulation (which arises from the aorta and intercostal arteries with high pressure) supplies a small proportion of the pulmonary tissue in mice compared to humans. These differences seem of importance when studying SAVI, where the vascular phenotype is predominant and endothelial cells may be a key player of lung disease [69]. Secondly, STING protein differs between humans and mice [70]. In addition, STING-deficient mice exhibited respectively an autoimmune profile associating splenomegalia with high levels of auto-antibodies and an expansion of inflammatory myeloid and denditric cells [71], and an increased number of specific autoreactive CD8+ T cells [72]. This is discordant with the autoimmune component observed in SAVI [52] and is indicative of the challenge in using mouse models in SAVI.

Considering these data and the limited access to human pulmonary samples, innovative models are needed to improve our understanding of SAVI. The use of induced pluripotent stem cells (iPSC) derived from SAVI patients could address this question [73]. Indeed, iPSC could subsequently be differentiated in relevant intrinsic lung cells, such as type II alveolar epithelial cells [74,75], endothelial cells, and also alveolar macrophages. Of interest, type II alveolar epithelial cells were shown to trigger a rapid and broad inflammatory response—including IFN production—in SARS-CoV-2 infection [76] and take part in lung fibrosis development [77]. Endothelial cells also seem particularly relevant, considering the vascular phenotype observed in SAVI patients [9]. Lung macrophages represent another population of interest, given their role in coordinating immune responses against airway microbes and pulmonary barrier integrity, and their contribution to lung fibrosis [78,79].

To further understand the complex architectural defect associated with *STING1* gain-of-function mutations, iPSC could be used to generate lung 3D organoids from SAVI patients [80]. Lung bud organoids accurately recapitulated the multi-lineage differentiation of lungs [81], thereby representing a model for early onset lung diseases [82], and the pulmonary maturation steps [81], making it also a useful tool to understand pulmonary fibrosis [80].

## 4. STING-Mediated Lung Disease beyond SAVI

### 4.1. COPA Syndrome

COPA syndrome is a recently described monogenic auto-inflammatory disease in which affected patients also present with severe ILD [25] and type I IFN overexpression [83]. The clinical overlap with SAVI suggested that constitutive STING activation was also central to the pathogenesis [24]. Indeed, dominant negative mutations in *COPA* were shown to lead to a constitutive localization of STING to the Golgi, where it was activated, due to disruption of STING enrollment in COP-I vesicles [7,26,27]. Interestingly, in the broad spectrum of type I interferonopathies, only SAVI and COPA syndrome display a severe lung phenotype, suggesting that STING dysfunction, rather than type I IFN overexpression per se, could be the cornerstone of the lung disease.

### 4.2. Dermatomyositis and Systemic Lupus Erythematosus (SLE)

The definition of type I interferonopathies can be extended to all pathologies in which a type I IFN overexpression has been found to be relevant to the symptoms. With these considerations, some autoimmune diseases could be classified as polyfactorial type I interferonopathies. Interestingly, patients with SLE [84] and dermatomyositis [85], specifically with anti-MDA5-positive antibodies, can present a strong IFN signature and a potentially lethal ILD [86]. Some of these patients have been reported to be improved by JAK inhibitors [87,88]. Of interest, STING activation by DNA containing extracellular vesicles seemed to drive inflammation in dermatomyositis [89]. These data suggest that STING pathway might be central to the pathophysiology of the pulmonary involvement in these more common diseases and that deciphering the lung pathology in SAVI could be relevant to them.

### 4.3. Beyond Type I Interferonopathies

As highlighted in this special issue, STING biology plays a key role in numerous pulmonary diseases [90]. In exposure disease such as silicosis, DNA liberated during silica-induced cell death activate STING pathway, thereby driving the lung inflammation [91]. It has also been suggested that self-DNA sensing through STING could be relevant to other more frequent lung diseases, such as cystic fibrosis, chronic obstructive pulmonary disease [92], idiopathic pulmonary fibrosis [93], or asthma.

## 5. Current and Future Therapies

### 5.1. JAK Inhibitors

Based on the assumption that increased type I IFN signaling represents a driver of pathology in type I interferonopathies, attention has focused on the use of Janus kinase (JAK) inhibitors. After activation, type I IFNs bind to their unique receptor (IFNAR1/2), which subsequently activates the JAK/signal transducer and activator of transcription (STAT) pathway. In this context, JAK inhibitors have been evaluated in monogenic and polyfactorial type I interferonopathies [7]. In SAVI, JAK inhibitors efficiently blocked STAT1 phosphorylation in B cells and T cells [9]. The first use of ruxolitinib, a selective JAK1/2 oral inhibitor, in patients was reported in 2016 in three affected children [54]. A positive effect was noted in patients, including on the lung status, as ground glass opacities decreased and pulmonary function tests improved. Several other reports reported global improvement under JAK1/2 inhibitors (ruxolitinib or baracitinib) in SAVI [83,94,95,96]. This was also confirmed in our longer-term follow-up cohort of eight patients treated with ruxolitinib, in which five patients improved their pulmonary radiological and functional parameters [58]. Among three patients with severe lung disease before JAK inhibition, one presented a mild improvement, however fibrosis progressed in the other two, who eventually required lung transplant. Their outcome emphasizes that severe lung disease is poorly responsive to treatment, warranting further therapeutic strategies and highlighting the need to detect pulmonary involvement at an early stage to avoid progression to end-stage respiratory failure.

Use of JAK inhibition in SAVI raises several questions. Pharmacokinetics (PK) and pharmacodynamics (PD) are highly variable, so the dose must be adapted to weight and renal function and repeated doses are often needed in younger children [97]. The type of organ involvement must also be taken into account as, for example, JAK inhibitors do not fully pass the blood–brain barrier [98]. Finally, our experience tends to favor the use of JAK1/2 inhibitors, such as ruxolitinib or baracitinib, over JAK1/3 inhibitors [99]. In SAVI, the main reported side effects were infectious (shingles, viral respiratory infections, rotavirus enteritidis, and aspergilloma in lung cavities). Papillary edema and ruxolitinib discontinuation syndrome were also reported [54,58]. Selective JAK inhibitors with a more specific anti-JAK-1 (e.g., filgotinib and upadacitinib [100]) activity could be promising to narrow the activity and improve safety, but inhibition of JAK2, by partially blocking NF-kβ pathway, could be relevant in SAVI [101].

### 5.2. Monoclonal Antibodies to Type I IFN Receptors

In the context of IL1-mediated autoinflammatory diseases, the use of IL1beta blockade has significantly improved the management of patients [102]. With this in mind, since type I IFNs seem to play an important role in SAVI pathogenesis, monoclonal antibodies blocking IFN alpha itself or IFNAR may be a valuable therapeutic option. Anifrolumab, a human monoclonal antibody to IFNAR1, has been developed and evaluated in SLE, considering the pathogenic role of type I IFN in this context [103]. After encouraging results from phase III trials [103,104], the Food and Drug Administration has recently approved Anifrolumab for SLE patients [105]. To our knowledge, no SAVI patient has benefited from this therapy yet.

### 5.3. STING Inhibitors

Considering the molecular mechanism of SAVI, a direct inhibition of STING might be a promising therapeutic option. Since 2018, several compounds acting on different steps of STING biology have been reported. Targeting the CDN binding site could allow STING inhibition. Li et al. recently identified a natural product, Astin C, that effectively binds to the CDN domain of STING dimers. In vitro, Astin C was able to inhibit type I IFN expression in *Trex1*^−/−^ bone-marrow-derived macrophages [106]. Another potential target of STING activation is the post-translational palmitoylation of STING that plays a crucial role in its activation. Several small covalent molecules inhibiting palmitoylation have been evaluated by the team of Andrea Ablasser [107] and others [108]. Of note, one of the compounds, the H-151 inhibitor, has been tested in PBMCs from one COPA patient and showed a reduction of *IFN-β* and ISG expression [109]. Finally, another molecule (ISD017), this time inhibiting ER–Golgi STING trafficking through STIM1, has been tested in the serum of SLE patients with high IFN-α levels [110]. Treatment with ISD017 decreased the concentration of the ISG protein CXCL10 in the serum of such individuals. Clinical translation of STING inhibitors will probably require a reasonable time frame and raises the question of potential infectious side effects and the appropriate choice of PK/PD in order to modulate STING activity rather than blocking it completely.

### 5.4. Antifibrotic Therapy

Antifibrotic drugs, e.g., nintedanib and pirfenidone, have been shown to be effective in controlling lung fibrosis progression in idiopathic pulmonary fibrosis [111]. In connective tissue diseases, such as systemic sclerosis, nintedanib is now part of the therapeutic arsenal available to treat fibrosing ILD. In the context of SAVI, in view of the early progression towards fibrosis, antifibrotic treatment may be a future treatment option, if approval for young children is obtained [112].

### 5.5. Lung Transplantation

Fibrotic lung disease in SAVI can lead to end-stage respiratory failure, requiring oxygen therapy, non-invasive ventilation, and sometimes lung transplantation. In our cohort, three patients—one young adult and two teenage children—underwent lung transplantation. One died quickly after lung transplant, another died of humoral rejection one year later, and the last one is still alive 2 years after the transplant [58]. In the literature, another SAVI patient underwent lung transplantation [113] at the age of 30 years old and died at 38 years of multiple organ dysfunction syndrome and fungal infection. So far, the post-transplantation follow-up is not sufficient to evaluate the risk of relapse after lung transplant. Addressing the question of the contribution of cells derived from the hematopoietic system in the pulmonary pathogenesis could help us in evaluating the risk of relapse after lung transplantation in SAVI.

### 5.6. Future Therapeutic Perspectives

Hematopoietic stem cell transplantation (HSCT) in SAVI has not been reported in the literature. As STING is expressed in the lungs and the lung pathology is possibly caused—at least in part—by intrinsic pulmonary cells, one can suppose that HSCT might not prevent lung inflammation and fibrosis. However, our current understanding of SAVI is too limited to not consider HSCT as a therapeutic option in this inborn error of immunity. The identification of a major hematopoietic component to lung pathogenesis would highlight the potential of HSCT as a treatment strategy. Similarly, the development of genome editing technologies is providing new possibilities in medical therapeutics [114], including in inflammatory disorders [115,116], and may be promising for SAVI patients.

## 6. Conclusions

Identification of SAVI emphasized the specific need to control STING homeostasis in human biology. Indeed, SAVI appears to be a unique model to study nucleic acid sensing through STING and its links to lung pathology. Even if type I IFNs are key drivers of pathology, as mirrored by the improvement of symptoms under JAK inhibitors, the pathogenesis of SAVI remains to be fully elucidated. Attention should be paid to NF-κβ activation, type III IFNs, autophagy dysregulation, and cell death. Concerning the lung pathology of SAVI, many questions remain: (i) Why does the fibrosis occur so precociously and severely in some patients and during adulthood with mild disease in others? (ii) To what extent is lung inflammation caused by intrinsic lung cells or by cells derived from the hematopoietic system? (iii) Why do SAVI patients have such a predominant lung phenotype with almost no neurological disease although STING is ubiquitously expressed? (iv) Is it possible to envision a similar lung pathogenesis between SAVI and COPA syndrome, the second Mendelian type I interferonopathy with predominant pulmonary involvement? Answering these questions would certainly enable us to further decipher the lung disease in more frequent pathologies in which type I IFN and STING play a key role, such as SLE, dermatomyositis, environmental exposure, or viral infection.

## Figures and Tables

**Table 1:** Main pulmonary findings in STING-associated vasculopathy with onset in infancy.

Clinical Symptoms
Cough
Tachypnea
Exertional dyspnea
Hemoptysis
Nail clubbing
**Radiological Findings**
Ground glass opacities
Septal thickening
Honeycombing
Crazy paving
Cysts
**Lung Function Tests**
Restrictive ventilatory impairment
Obstructive ventilatory impairment
Hyperinflation
Mixed pattern
**Broncho-Alveolar Lavage**
Alveolitis (lymphocytic, neutrophilic, or mixed)
Intra alveolar hemorrhage
Normal
**Histopathological Findings**
Fibrosis
Lymphoid follicles with germinal left organization
Inflammatory infiltrate
Alveolar hemorrhage
